# Survivor and parent engagement in childhood cancer treatment in Iran

**DOI:** 10.3332/ecancer.2021.1220

**Published:** 2021-04-19

**Authors:** Shirin Ahmadnia, Atena Kamel Ghalibaf, Saba Kamkar, Zahra Mohamadzadeh, Mithra Ghalibafian

**Affiliations:** 1 Allameh Tabataba`i University, Tehran 1489684511, Iran; 2 MAHAK Charity–Society to Support Children Suffering from Cancer, Tehran 1956993461, Iran; 3 MAHAK Paediatric Cancer Treatment and Research Centre (MPCTRC), Tehran 1956993461, Iran

**Keywords:** adolescent, physician–patient relationships, cancer survivors, parents, communication, engagement, Iran

## Abstract

In Iran (with a population of 83 million), 19,973 children were diagnosed with cancer from 2009 to 2019 (MAHAK Charity). This study was part of the International Society of Paediatric Oncology, Paediatric Oncology in Developing Countries Committee, ‘Patient, Family and Stakeholder Engagement Task Force’ engagement study in ten low- and middle-income countries in 2019. We documented childhood cancer survivors and parents’ experiences and quality of engagement, including perceptions and expectations, during their cancer treatment journey in Iran. Fourteen in-depth interviews and three focus group discussions were conducted by three Iranian qualitative researchers with 29 participants: recent adolescent/young adult survivors (12–20 years), long-term survivors (21–30 years) and parents [36–61 years (six mothers and four fathers)] from diverse socio-economic and ethnic backgrounds. Data were recorded, transcribed and analysed, and then translated from Farsi into English. Participants’ expectations included *inter alia* improved communication and information flow among the key stakeholders including doctors, nurses, parents and patients. A need for improved patterns of doctor–patient relationships and communication, more effective psychosocial support and the importance of peer support groups (for survivors and parents) were reported. Participants identified areas of satisfaction and dissatisfaction regarding their actual engagement and decision-making. The dominance of a medical-only approach instead of multidisciplinary approach in care, the shortage of psychosocial support, the lack of an integrated system for providing information and delivering a package of printed material about the treatment journey and the absence of patient navigator in childhood cancer care systems were among obstacles for successful engagement of patients and parents throughout their cancer journey.

## Introduction

According to a model created by Ward *et al* [1], childhood cancer incidence in 2015 was estimated to be 397,000 worldwide. Atun *et al* [2] predict that between 2020 and 2050 there will be 13,659,000 new cases of childhood cancer globally and 2,509,000 in upper-middle-income countries.

In Iran, an upper-middle-income country with a population of 83 million, 19,973 children were diagnosed with cancer within the last 10 years based on data from the Society to Support Children Suffering from Cancer (MAHAK Charity), corresponding to the findings in a study conducted by Shabani *et al* [3]. These statistics roughly represent national figures, since MAHAK supports almost all children with cancer throughout the country. According to a recent Iranian study, the crude incidence of childhood cancer (0–14 years) was 16.8 per 100,000 for males and 16.56 per 100,000 for females [4].

Iran borders seven countries: Iraq, Turkey, Armenia, Azerbaijan, Turkmenistan, Afghanistan and Pakistan. It is a multi-ethnic nation with major groups, including Persians (61%), Azerbaijanis (16%; Azeris) and Kurds (10%) [5]. It is estimated that there are approximately 1 million Afghan migrants and refugees living in Iran [6]. Iran’s Gini coefficient was 40.8 in 2017 [7] and the gross domestic product per capita in 2017 was USD 5,520.3 [8].

Children and adolescents with cancer in Iran are treated in 32 hospitals under the supervision of medical sciences universities and governmental medical centres across the country. Efforts have been made to create universal healthcare in the country, but it is not yet realised [9]. Childhood cancer treatment costs are covered by a number of healthcare insurance companies and non-governmental organisations (NGOs). According to a recent study in Iran, families with a child with cancer are generally referred to insurance organisations and NGOs for financial support since they face substantial financial challenges [10]. MAHAK Charity is an NGO founded in 1991, and supports all children (ages 0–16) with cancer in Iran.

Families of children/adolescents with cancer worldwide face multiple difficulties, endure negative experiences and face many challenges. Edwards and Greeff in South Africa concluded that throughout the process of treatment, poor standards of information delivery and lack of cancer knowledge provision by the healthcare system personnel and a lack of patient-centred care have been reported to be of great concern for parents [11]. In Romania, researchers found that ‘provision of information is a fundamental step towards shared decision-making. It enables minor patients and families to frame personal values when making decisions’ [12]. The importance of an efficient doctor–patient relationship is well documented in the literature on health communications and sociology of medicine [13]. A study in Malawi reported that guardians felt reluctant to ask the nurses and doctors questions, because they were afraid that they would be told off or they were shy [14]. On the contrary, according to a study in the US, parents whose children were at the end of life were reported to be quite satisfied with the availability of healthcare providers and the adequacy of information received about their child’s diagnosis and symptom management [15].

There are two sides of the therapeutic relationship, patients and their parents and healthcare professionals (doctors mainly, but also nurses, psychologists and social workers). In the current study, we focused only on one side – patients and their parents. This study was conducted as part of the International Society of Paediatric Oncology, Paediatric Oncology in Developing Countries Committee, ‘Patient, Family and Stakeholder Engagement Task Force’ study in low- and middle-income countries in the summer of 2019. One common finding across all project countries was communication issues between healthcare personnel and parents and patients, among family members including extended family members. The authors of this article documented survivors and parents’ experiences of treatment engagement, perceptions and expectations, level and quality of engagement, particularly communication, during a child/adolescent’s cancer treatment journey in Iran.

## Methods

This qualitative study included a total of 29 participants representing: a) ‘adolescent and young adult survivors’, b) ‘long-term survivors’ and c) ‘parents’ from across the country. We held three focus group discussions (FGDs) and 14 in-depth interviews (IDIs). Three FGDs were held with a total of 15 participants divided as follows: a) seven adolescent and young adult survivors, b) three long-term survivors and c) five parents. Fourteen IDIs with additional participants (distinct from those in the FGDs) were conducted with the following: a) five adolescent and young adult survivors, b) five long-term survivors and c) four parents (see Table 1 for eligibility criteria).

The authors defined a sampling framework to achieve sociocultural diversity among a range of ethnicities in Iran. MAHAK Charity supports families of children with cancer in all 40 centres across Iran. Participant recruitment and access was realised through the MAHAK Charity database and archives of patient records using administrative support and reflecting MAHAK Charity’s nationwide coverage. (Table 2 shows the socio-demographic characteristics of the study participants.)

Potential participants from the capital city and other provincial cities were solicited to travel to the interview site in Tehran by MAHAK’s social work department; participants’ transport costs were provided by MAHAK. All participants agreed to take part in IDIs and FGDs and signed a participant consent form in Farsi after being made aware of the study’s purposes.

All IDI and FGDs were recorded, transcribed in Farsi and content analysis was carried out to identify codes by three expert qualitative researchers (all authors of this paper: SA, AK, and ZM). Data analysis in this study focused on the survivors and parents’ engagement during cancer treatment.

## Results

Participants were recruited from diverse ethnicities, regions and genders to represent the multicultural context of Iranian society. (See Table 2 for the socio-demographic characteristics of the study participants.)

The results are categorised in to four main themes: 1) participant expectations, 2) nature and extent of actual engagement, 3) degree of satisfaction with the nature and level of engagement, and 4) degree of engagement affecting overall care experiences.

### Participant expectations

The parents and survivors’ expectations from the medical team were described and characterised as their ‘ideal’ doctor/nurse and parent/patient relationship. The participants expected doctors to be good listeners, medical experts and experienced, responsive, having friendly attitudes, kind, sincere, accessible, patient, providing hope, up to date and sensitive to the pain and problems of their patients. Their expectations of nurses were to be expert (especially with regard to phlebotomy), experienced, with a friendly attitude, kind, sincere, understanding, patient, with good manners, giving hope and encouragement. Patient 13, a 28-year-old survivor of Hodgkin disease said:

Doctors need to spend much more time [with their patients]. They should explain everything well for the patient. They should give the information in simple understandable words. Doctors are looking down on patients. At last, much is depending on his experience and expertise.

Patient 14, a 21-year-old male who survived rhabdomyosarcoma, said, ‘the nurses treated children kindly. They were patient enough for this job. Nurses should be kept responsible for what they do’. Parent 4, a 44-year-old father of a child who survived glioma mentioned: ‘the family expects the care team to guide them more on what they should do and how to deal with the problems’.

### Nature and extent of actual engagement

Decision-making on treatment options, symptom management and/or pain management options included the parents’ lack of ability and control to decide on changes in the treatment or to change the hospital where the child was being treated. As parent 6, a 39-year-old mother of a survivor of non-Hodgkin lymphoma, said: ‘We had to rely on the doctor’s decisions. We could only decide about financial issues. We were not supposed to get involved in other matters’. Most of the parents were inactive when it came to making decisions and were receptive towards their child’s doctors’ decisions. Parents mentioned feeling powerless. They even made reference to when medical staff made wrong decisions that negatively impacted their child. The following quotation shows the attitude belonging to most parents. Parent 3, a 36-year-old father of a survivor of neurofibromatosis, stated: ‘We believed that we shouldn’t bother doctors and not to cause them headaches. My wife and I believed in and had faith in our doctor. We accepted whatever he told us because he had graduated and was an expert in this field so we trusted him’.

It was common for parents to avoid disagreement or protest when communicating with healthcare professionals and no participant reported taking legal action against a healthcare professional even when they had witnessed something going wrong. Patient 16, a 24-year-old female survivor of acute lymphoblastic leukemia (ALL) told the following story:

Once, they injected me a wrong medicine, then I complained afterwards that I got a terrible stomach ache. They did not take my complaint seriously since I was only a kid at the time. Later however they found out that due to that mistake, my kidneys failed and they transferred me to ICU (The interviewer asked if her parents protested against the person in charge in the hospital following that occurrence and she said no, they never even thought of something like that).

Some parents reported they were able to make decisions. One example of a rare active decision-making was Patient 1, a 14-year-old survivor of ALL who spoke about a time his mother took self-reliant action:

First, I was under the supervision of a doctor who cared for me a lot. After a while, though, my mother searched a bit, got consultation from other people and found out another doctor in the same hospital was more of an expert. She decided to make a change in who to treat me. I then got transferred to a second doctor. Having learned this change, the first doctor got upset and told me: ‘This is not fair. You are being unkind to me. I liked you so much and I would rather have you under my supervision. Why should your mother turn to the other doctor!’

Parent 10, a 38-year-old mother of a survivor explained her bad experience when requesting a CT for her child at the time and her reaction:

I told the doctor: Could you please take a CT scan of my child’s lung? ‘No Madam, why are you disturbing your child [by demanding this]? There is nothing to worry about’. I insisted: If there is nothing to worry about, then please do as I wish because it will be a relief to me. He finally reluctantly agreed with the CT scan after I insisted, and it was only then that we realised there was actually a mass in my child’s lung...’.

### Necessity of a medical/psychosocial supportive care team

Several respondents spoke about their difficulty in accessing doctors, nurses and the supportive care team. They expressed that these healthcare professionals were not reachable or were inaccessible. Patient 13, introduced earlier, stated:

My mother usually had to run after doctors and it was a tough situation especially when I was going through a difficult phase that is, when my illness relapsed. As an example, I remember they came to the ward to visit us, but the doctors could not stay long being too busy. My mother had to run after them in the stairs to be able to pose her questions.

Patient 15, a 24-year-old male survivor of a small round cell tumour, on the contrary, spoke positively about the psychosocial team’s good accessibility (i.e., psychologist and social worker). ‘Social workers were there to support mothers. They came to visit the mothers to encourage them. They made sure the mothers fully understood the situation’.

However, for communication of medical information and education about the child/adolescent’s cancer, particularly during diagnosis, parents mentioned that the doctors were not completely transparent. The doctors used jargon while delivering information, which had a negative effect on the quality of communication. Thus, parents described using tactics to compensate the lack of information and education, e.g., tactics such as searching on the Internet or using peer group consultation. According to Parent 9, a 35-year-old mother of a survivor of neuroblastoma:

The doctor should be transparent and clear in announcing the illness. We usually do not understand what doctors say [medical jargon]. A doctor should talk to us in simple words according to and based on our level of knowledge and understanding.

Parent 1, a 38-year-old mother of a survivor of neuroblastoma explained:

We begged whoever [personnel] we had access to, behind the operation room, in order to ask for any information such as the surgeon’s words about the operation and what was going on with our child’s health there. Sometimes we took a copy of the reports being transferred from one department to the other. Most of the times, however, the information we got was of no use, because the terminology used was complicated and in English, so we could not understand anything! We therefore, usually asked other parents with earlier similar experiences, for example, we asked them ‘what does “calcification” mean?’ At the time, not many people had smart-phones to be able to search easily for unknown things to come to an answer.

On the other hand, some parents stated that they were intentionally avoiding detailed information due to their fear of diagnoses of cancer or because of denial. Parent 5, a 61-year-old father of a survivor of medulloblastoma explains:

Whenever we asked any questions the doctor did answer us as much as we wanted. But we often did not inquire ourselves; i.e., we were afraid of asking too much because in that case the doctor had to tell us much of the details and the negative facts which we would rather not hear or learn.

The parents’ sociocultural background impacted their communication with the medical team in terms of the discrimination they experienced as Patient 17, a 28-year-old female survivor of rhabdomyosarcoma mentioned:

According to the city/town from which patient’s family came and/or their educational background, they received different levels of attention and services from the care team [that is, discriminatory]. Doctors had a better behaviour towards a child whose parents were e.g. lawyers, or they didn’t care much when explaining things to parents who were less educated or from a lower social class; Doctors assumed that those parents cannot understand much, therefore they did not speak to them with details or for long.

### The degree of satisfaction with the nature and level of engagement

The dissatisfaction with the medical team was mostly reported due to a lack of information provided by doctors about treatment procedures and/or disease. However, it was also related to how doctors broke bad news and/or nurses and doctors not being responsive (answering questions) or clear enough. Parent 10, a 38-year-old mother of a survivor of ALL related a negative experience:

I told my doctor that I am really angry with you. You are not telling me anything. I do not care if sometimes you play with my child, make humour or you are being nice to my child. What matters to me is to know what’s really going on with my ill child! The doctor replied that I am not supposed to put you in a stressful situation by disclosing the details of the treatment.

A survivor of osteosarcoma, 17-year-old Patient 8, shared her positive experience:

There was a nurse who brought me homemade food to cheer me up. Sometimes she gave me gifts, some hair pins or lip balms. I was eagerly waiting to see her. Some of nurses helped me with my school home-works and let me sit in their room with them to have fun. They were kind to me. This nice behaviour helped me not to think of hospital as a bad and horrible place.

Parent 3, introduced earlier, shared a story about experiencing discrimination because of his Afghan ethnicity: ‘Since we are Afghan migrants, we were not treated respectfully and in a decent manner in a hospital. If we were Iranians, they would not disrespect us that way. However, at the hospital in Tehran we received respect and there were no discriminations’.

Financial aspects of treatment are a main concern for the families. Families and survivors were happy with the financial support they received from NGOs and therefore experienced the elimination of a financial relationship with the doctors. Patient 15, introduced earlier, said:

We heard that adult patients with cancer had to sell everything they owned to pay the treatment costs. There were ill people leaving the treatment due to not being able to pay the costs. However, this charity offers financial support for treatment and even provides financial support for university tuition fees for recovered patients.

### Degree of engagement affecting the overall care experience

Parents and survivors spoke about the care experiences they had had with the medical team (i.e., doctor/nurses) and some mentioned the doctor’s lack of sympathy and empathy. Patient 3, a 12-year-old female survivor of medulloblastoma said, ‘I would have liked to open up to my physician and enjoy his sympathy. If the physician would have talked to me, giving me hope, I would have recovered earlier than I did’. Patient 19, a 30-year-old survivor of ALL mentioned: ‘Doctors suppose emotional support and showing affections are not considered their duties. This is why they kept their distance from us’.

Patient 18, a 23-year-old male survivor of a central nervous system tumour spoke about the positive and negative relationships with the psychosocial support team and shared the following: ‘Parents need to be trained by psychologists to learn how to treat their children throughout the stages of the illness. This could have positive impact and influence children’s mental health’.

Parent 2, a 41-year-old mother of a survivor of a primitive neuroectodermal tumor (PNET), spoke positively about the multidisciplinary team approach in treatment journey:

Physicians, nurses, social workers and psychologists along with each other and with a good cooperation can achieve optimal outcomes during treatment. We understand that nurses may not be capable of practicing some stuff, such as showing kindness and necessarily being companionate (due to work overload), therefore we need social workers who could be nice to children and talk to them. And as for parents, the presence of psychologists would be really helpful and effective.

The parents mentioned the necessity for an integrated system for providing information and delivering a package of printed material for parents, right in the beginning of the treatment journey and throughout (A–Z). They wanted the company of someone in the healthcare centre just to guide them step by step in every phase. Patient 4, a 20-year-old female survivor of a germ cell tumour, explained:

There was no plan or systematic anticipation to inform and teach families about the illness details and the treatment process at the beginning of the hospitalisation. I suppose this is very important. It could at least ease and comfort families a little bit. It’s important to inform families, e.g. about the side effects of the treatment beforehand.

## Discussion

For the successful engagement of parents and patients with the medical team throughout the cancer treatment journey, our participants felt the need to be fully informed about the disease and treatment procedure details in simple understandable words and asked for a better relationship with the caregiving team. They believed this would build a positive and effective communicative relationship with the medical team, especially since doctors play a crucial role in this, despite the fact that it is often neglected in the country. Zamanzadeh *et al* [16] investigated factors affecting the communication between patients with cancer and nurses in Iran. In order to communicate with cancer patients effectively, they found that changes in the ‘philosophy and culture of the care environment’ and focusing on ‘holistic and patient-centred’ care are essential, Effective communication was also found to be essential for paediatric cancer treatment in a study conducted in the US, where ‘clear and empathic delivery of diagnostic and prognostic information positively impacts the ways in which patients and families cope’ [17]. These findings are similar to our study results.

The lack of proper communication between parents and patients and the medical teams, the dominance of a medical-only approach instead of a multidisciplinary approach in childhood cancer care and the absence of paediatric psychology support were found to be among the most important obstacles for the successful engagement of patients and parents throughout their cancer journey. These issues were also found to be problematic in India, where Singh *et al* [18] identified ‘several barriers to communication such as paternalism in medicine, inadequate training in communication skills, knowledge of the grieving process, special issues related to care of children, and cultural barriers’. In Kenya, Njuguna *et al* [19] emphasised that paediatric oncology doctors needed to improve their communication skills. In regard to adolescents with cancer in Switzerland, Essig *et al* [20] found that their communication needs are rarely met. They noted that differential expectations regarding good communication by doctors and nurses should be considered when developing communication skills courses for oncology professionals.

A multidisciplinary approach was suggested by our participants, wherein they mentioned the need for the presence of a psychologist in addition to the social worker to sit in the room when the doctor broke the bad news of their child’s cancer diagnosis. The parents stated that this interdisciplinary harmony needs to be developed and reinforced throughout the treatment journey. They stated that they were happy with the doctor’s expertise, but for psychological pain and fear, they needed a specialist’s help and support; this is something a psychologist would provide, but the doctors usually are not capable of or do not care about the same. Keramatinia *et al* [21] in Iran concluded that a multidisciplinary approach *inter alia* was associated with lower risk of mortality and improved survival of children with various cancer diagnoses. Carlson *et al* [22] also found that ‘a multidisciplinary approach supported clinical efficiency and fostered seamless patient-centred care’ for childhood cancer survivors in the US.

Parents required clear and consistent explanations about their child’s disease and treatment procedure. This need is partly recognised and mentioned in ‘the patient’s bill of rights’, which is usually written on a paper in a frame hanging on a wall at the hospital for the patients to see, but is not practiced actually or effectively. As a solution or to substitute the lack of adequate information and communication, which would increase their engagement in the treatment, the participants in our study suggested the provision of peer support groups for parents and patients. Some of them actually played and still play such a supportive and informative role for new patients in the hospitals. In this way, they compensate for what is lacking in the current (inadequate) relationship among doctors and patients/parents. For example, experienced parents would interpret the doctors’ words for the parents of a sick child just hospitalised, and/or explain what parents should do whenever the child’s condition became acute or how to manage pain etc. These peers would give advice based on their own experiences.

Therefore, when we asked parents and survivors for their feedback on potential areas for a better engagement, they suggested forming committees consisting of peer groups with the same experiences. They spoke of the formation of groups such as survivor or parent support committees. This is congruent with the findings of a study by Smith *et al* [23] in the US, who referred to the need for social support groups among adolescents and young adults with cancer. Swedish researchers Ljungman *et al* [24] also suggested ‘facilitating access to social support systems as an important aspect of psychological help to parents of children on cancer treatment’.

The participants in our study also recommended appointing a position in the hospital for a person to be in charge of guiding and accompanying new diagnosed patients and families through wards of hospitals (i.e., patient navigator). Schroeder *et al* [25] stated that ‘oncology patient navigation programmes have been successfully implemented in hospitals throughout the US enhancing the quality of cancer care’. A pilot patient navigation programme in Tanzania was established by those researchers ‘to assist patients and their families with care coordination throughout the diagnosis and treatment process’. Their results suggest that even in a resource-limited setting, navigation programmes are a reasonable strategy for tackling barriers to care delivery and treatment abandonment, thus improving survival.

A comprehensive information package available at the beginning of the childhood cancer treatment journey (following a cancer diagnosis) could be provided for parents and patients as guidance on the child/adolescent’s disease and relevant treatment procedures. The information would cover physical, mental and social guidelines, tips and advice for frequently asked questions and highlight the parents’ role for improved involvement and engagement during the treatment journey. This would not require high costs and budgets could be covered under the routine treatment costs, while exploiting the support provided by social workers in the hospitals.

Elimination of the financial relationship between families and doctors, which is seen as an important factor for a better engagement of the families with the hospital medical teams (to include all potential patients and not only the higher socio-economic classes), is already on the agenda of the Ministry of Health. This has been partially achieved through the implementation of the new Health Reform Plan launched a few years ago with the purpose of realising social justice and reducing families’ out-of-pocket health and care costs. Rarely hospitals in Iran have realised this approach. The participants in this study who had accessed this opportunity and received free services have expressed their high satisfaction about this.

## Conclusion

As we explored the engagement quantity and quality of parents and survivors, we realised that parents were concerned about the lack of proper communication not only with physicians, but also with nurses, psychologists and social workers, and expressed a need for direct contact and dialogue. This will most likely require training in communication skills during diagnosis, investigations and treatment. In Iran, it is not common among healthcare professionals to function as a multidisciplinary team, which according to our findings is assumed to be necessary by patients with cancer and their parents. Implementation of a multidisciplinary approach in the childhood cancer treatment journey with an emphasis on the presence of psychosocial supportive team alongside the medical care team, especially when breaking bad news, is concluded to be necessary. This approach is feasible in Iran, with simple revisions in healthcare policies made at the level of the Ministry of Health guidelines and protocols or in every hospital if the director could be convinced that this would result in better healthcare outcomes. Written material for families about childhood cancer is another important communication tool for improved engagement and should be developed.

Continued exploration of patient and parent engagement during childhood cancer treatment across Iran is warranted. Implementation of participant recommendations and evaluation of these changes will reveal the effectiveness of the changes in practice. Patient and parent engagement is a critical part of childhood cancer care and must be prioritised and respected.

## Funding

The authors would like to thank MAHAK Charity for its financial support for the research project, administrative support and provision of access to the participants for IDIs and FGDs.

## Conflicts of interest

Saba Kamkar and Zahra Mohamadzadeh hereby declare their association as full-time employees of MAHAK﻿ Charity–Society to Support Children Suffering from Cancer and Mithra Ghalibafian hereby declares her association as full-time employee of MAHAK Paediatric Cancer Treatment and Research Centre (MPCTRC). Shirin Ahmadnia and Atena Kamel Ghalibaf, who voluntarily participated in this study, hereby declare that they have no conflict of interest of any kind.

## Figures and Tables

**Table 1.** Sample characteristics of participant eligibility criteria and study sample.

Groups of participants	Potential intended samples	Study samples
Adolescent/young adult survivors	Adolescent and young adult survivors who had completed treatment <2 years	12 recent survivors (12–20 years)
Longer-term survivors	Longer-term survivors (all treatment being completed before the age of 18) and >5 years incident free	7 longer-term survivors (21–30 years)
Parents	Parents as care givers during their child’s treatment occurring < 5 years	10 parents (36–61 years)

**Table 2.** Socio-demographic characteristics of the study participants.

Adolescent/young adult survivors					
	Participant pseudonym	Gender	Age in years	Ethnicity	Iran region
1.	Patient 1	Male	14	Persian	Center
2.	Patient 2	Male	17	Persian	Center
3.	Patient 3	Female	12	Arab	South
4.	Patient 4	Female	20	Kurd	Center
5.	Patient 5	Male	20	Azari	Center-rural area
6.	Patient 6	Male	17	Persian	Center
7.	Patient 7	Male	15	Lur	West
8.	Patient 8	Female	17	Persian	Center
9.	Patient 9	Female	15	Persian	Center
10.	Patient 10	Male	16	Persian	Center
11.	Patient 11	Female	15	Persian	South
12.	Patient 12	Female	13	Afghan	Center
**Longer-term survivors**					
1.	Patient 13	Male	28	Persian	Center
2.	Patient 14	Male	21	Kurd	Center
3.	Patient 15	Male	24	Azari	Center
4.	Patient 16	Female	24	Azari	Northwest
5.	Patient 17	Female	28	Azari	Center
6.	Patient 18	Male	23	Persian	Center
7.	Patient 19	Male	30	Persian (Mazani)	North
**Parents**					
1.	Parent 1	Female	38	Azari	Center
2.	Parent 2	Female	41	Persian	Center
3.	Parent 3	Male	36	Azari	Center
4.	Parent 4	Male	44	Persian	Center
5.	Parent 5	Male	61	Afghan-immigrant	Center-rural Area
6.	Parent 6	Female	39	Persian	Center
7.	Parent 7	Female	44	Azari	Center
8.	Parent 8	Female	33	Persian	East
9.	Parent 9	Female	35	Azari	Center
10.	Parent 10	Female	38	Persian	Center
